# Titration and characteristics of pressure-support ventilation use in Argentina: an online cross-sectional survey study

**DOI:** 10.5935/0103-507X.20200013

**Published:** 2020

**Authors:** Joaquin Pérez, Javier Hernán Dorado, Ana Carolina Papazian, Maricel Berastegui, Daniela Inés Gilgado, Gimena Paola Cardoso, Cristian Cesio, Matías Accoce

**Affiliations:** 1 Sanatorio Anchorena de San Martín - San Martín, Buenos Aires, Argentina.; 2 Sanatorio Colegiales - Buenos Aires, Argentina.; 3 Hospital de Quemados - Buenos Aires, Argentina.

**Keywords:** Health care surveys, Respiration, artificial, Interactive ventilatory support, Positive-pressure ventilation, Intensive care units, Pesquisas sobre Serviços de Saúde, Respiração artificial, Suporte ventilatório interativo, Respiração com pressão positiva, Unidades de terapia intensiva

## Abstract

**Objective:**

To identify common practices related to the use and titration of pressure-support ventilation (PC-CSV - pressure control-continuous spontaneous ventilation) in patients under mechanical ventilation and to analyze diagnostic criteria for over-assistance and under-assistance. The secondary objective was to compare the responses provided by physician, physiotherapists and nurses related to diagnostic criteria for over-assistance and under-assistance.

**Methods:**

An online survey was conducted using the Survey Monkey tool. Physicians, nurses and physiotherapists from Argentina with access to PC-CSV in their usual clinical practice were included.

**Results:**

A total of 509 surveys were collected from October to December 2018. Of these, 74.1% were completed by physiotherapists. A total of 77.6% reported using PC-CSV to initiate the partial ventilatory support phase, and 43.8% of respondents select inspiratory pressure support level based on tidal volume. The main objective for selecting positive end-expiratory pressure (PEEP) level was to decrease the work of breathing. High tidal volume was the primary variable for detecting over-assistance, while the use of accessory respiratory muscles was the most commonly chosen for under-assistance. Discrepancies were observed between physicians and physiotherapists in relation to the diagnostic criteria for over-assistance.

**Conclusion:**

The most commonly used mode to initiate the partial ventilatory support phase was PC-CSV. The most frequently selected variable to guide the titration of inspiratory pressure support level was tidal volume, and the main objective of PEEP was to decrease the work of breathing. Over-assistance was detected primarily by high tidal volume, while under-assistance by accessory respiratory muscles activation. Discrepancies were observed among professions in relation to the diagnostic criteria for over-assistance, but not for under-assistance.

## Abstract (es)

**Objetivo:**

Identificar las prácticas habituales de uso y titulación del modo presión soporte (PC-CSV - *pressure control continuous spontaneous ventilation*) en pacientes bajo ventilación mecánica y analizar las formas de reconocimiento de sobreasistencia y subasistencia. Secundariamente, comparar las respuestas según profesión en relación al diagnóstico de sobreasistencia y subasistencia.

**Métodos:**

Se realizó una encuesta online utilizando la herramienta *Survey Monkey*. Se incluyeron a médicos, enfermeros y kinesiólogos de Argentina que tuvieran acceso al uso de PC-CSV en su práctica habitual.

**Resultados:**

Se recolectaron 509 encuestas desde octubre a diciembre 2018. El 74,1% de ellas correspondió a kinesiólogos. Un 77,6% refirió utilizar PC-CSV para iniciar la fase de soporte parcial. Un 43,8% selecciona el valor de presión de soporte inspiratorio basándose en volumen corriente. El principal objetivo de la selección de PEEP fue disminuir el trabajo respiratorio. El volumen corriente alto fue la variable primordial de detección de sobreasistencia, mientras que el uso de músculos accesorios fue la más elegida para subasistencia. Se observaron diferencias entre médicos y kinesiólogos en relación a las formas de detección de sobreasistencia.

**Conclusión:**

El modo más utilizado para la fase de soporte parcial es PC-CSV. La variable más elegida para titular la presión de soporte inspiratorio es volumen corriente y el principal objetivo de la PEEP es disminuir el trabajo respiratorio. La sobreasistencia es detectada prioritariamente por un volumen corriente elevado, mientras que la subasistencia mediante el uso de músculos accesorios. Se halló diferencias entre profesiones en relación a los criterios de detección de sobreasistencia.

## INTRODUCTION

The implementation of analgesia-based sedation protocols that promote the patients to keep awake, calm and comfortable have led to an early need to optimize patient-ventilator interactions in intensive care units (ICUs).^(1-3)^ Consequently, the use of spontaneous ventilatory modalities during withdrawal of invasive mechanical ventilation (IMV) has increased considerably.^(4,5)^ In particular, pressure-support ventilation (PC-CSV - pressure control-continuous spontaneous ventilation) has gained ground in relation to other partial support modalities, such as synchronized intermittent mandatory ventilation, mainly due to the negative effects of the latter in terms of ventilator dyssynchrony and delayed weaning.^(6-8)^ In the latest research cohort reported by Esteban et al., the subjects remained 237 days (23.7%) ventilated in PC-CS V for each 1000 days of IMV. Therefore, PC-CSV was the most widely used ventilatory mode after the sixth day of IMV.^(3)^

Despite its increasing popularity, there are no clear recommendations on how to program PC-CSV, especially regarding inspiratory pressure support (PS) and positive end-expiratory pressure (PEEP) settings.^(9,10)^ Previous investigations have focused on evaluating the ability of single variables such as occlusion pressure in the first 100 msec (P_0.1_), respiratory rate (RR), tidal volume (Vt) and use of accessory respiratory muscles (AMs) to estimate the work of breathing (WOB) during different levels of PS.^(9,11-13)^ However, there is scarce information describing how frequently these variables are used to select PS in daily practice. Additionally, there is no consensus on the goals of PEEP in PC-CSV, nor have we widely accepted definitions of “over-assistance” and “under-assistance”, two frequent clinical situations whose complications have been extensively reported.^(9,10,14)^

The purpose of this study was to identify common practices related to the use and titration of PC-CSV in patients under IMV and to analyze diagnostic criteria for over-assistance and under-assistance. The secondary objective was to compare the responses provided by physicians, physiotherapists and nurses related to diagnostic criteria for over-assistance and under-assistance.

## METHODS

An observational, analytical and cross-sectional online survey was conducted.

The survey consisted of questions aimed at understanding the methodology of PC-CSV use and titration in the partial support phase of patients under IMV.

The development of the questions was carried out following recommendations from previous studies.^(15-17)^

A literature search was performed in the MEDLINE database using the terms “pressure support ventilation” AND “mechanical ventilation” AND “intensive care unit”. Based on the results, relevant articles in English or Spanish were identified, and a full text review was conducted of those that included information inherent to PC-CSV use and titration. Additionally, bibliographic references were consulted to expand the selection of relevant information. In addition, six health professionals (three physiotherapists, two physicians and one nurse), specialists in intensive care were consulted regarding variables and relevant questions to include in the survey. Subsequently, the information was summarized, an initial version of the survey was developed and later reviewed by the authors of the study along with one physiotherapist and one intensivist physician. As a result of these interviews, the second version consisting of 19 items was constituted and approved by consensus. This version was evaluated in a pilot test including 10 participants who completed the survey and reported the degree of clarity of the statements.

After completing the pilot test, the following corrections were made: a) items 7 and 16 were modified to accept multiple answers; and b) items 11, 12, 14 and 15 were rephrased. Afterwards, the final version of the survey was constituted (Appendix 1).

Physicians, nurses and physiotherapists who work in units located in Argentine with access to PC-CSV in their daily practice were included. A convenience sample was obtained from a database developed by the authors of the study.

The subjects were invited to participate using a non-probability sampling technique by sending the survey via email and through social networks (WhatsApp^®^, Twitter^®^ and Facebook^®^). The link was shared by three of the researchers, and if no response was obtained, a new email was sent every two weeks up to a maximum of three times. The invitation contained the objective of the study and a link to access the survey using the Survey Monkey^®^ tool (https://es.surveymonkey.com). The responses were archived in a database provided by the website used and subsequently downloaded to a Microsoft Excel® spreadsheet from which the corresponding analyses were performed.

### Statistical analysis

The categorical variables are presented as absolute numbers and percentages and compared using the X^2^ test or Fisher’s exact test, as appropriate. IBM SPSS Macintosh, version 24.0 (IBM Corp., Armonk, NY, USA) was used for data analysis.

## RESULTS

**Figure 1:**
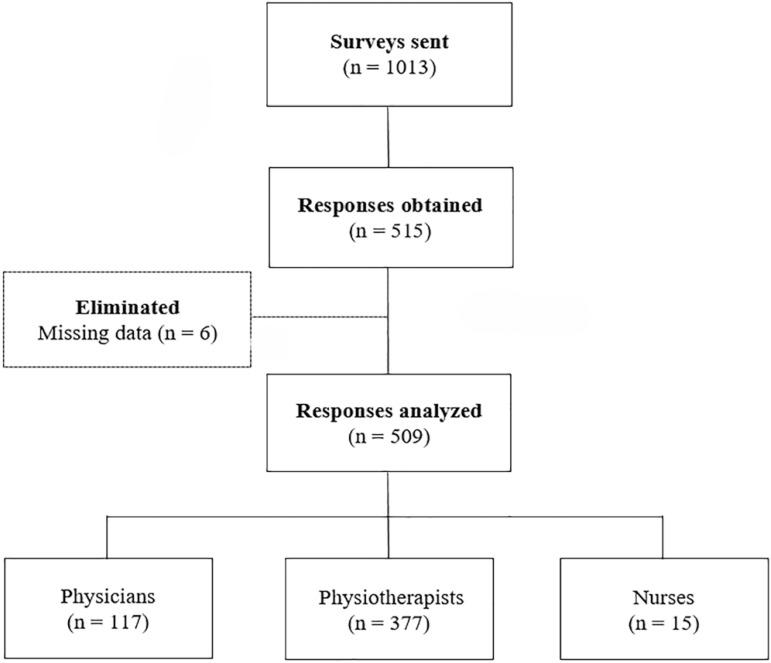
Flowchart.

During the period between October 8 and December 31, 2018, a total of 1,013 invitations were sent via email and WhatsApp^®^. The link was shared 5 times on Twitter^®^ and Facebook^®^. A total of 515 responses were obtained, and 6 were eliminated (1.2%) because of incomplete data, leaving 509 to be analyzed (Figure 1).

The response rate was 50.2%, and the majority of respondents were physiotherapists (74.1%) and subjects between 25 and 34 years old (50.7%) (Table 1).

**Table 1:** Demographic variables

Age (years)	
< 24	11 (2.2)
25 - 34	258 (50.7)
35 - 44	160 (31.4)
45 - 50	54 (10.6)
> 50	26 (5.1)
Profession	
Physiotherapists	377 (74.1)
Physician	117 (23)
Nurse	15 (2.9)
Experience in ICU (years)	
< 5	180 (35.3)
5 to 9	151 (29.6)
10 to 14	92 (18)
15 to 19	45 (8.8)
> 19	41 (8)
Type of institution	
Public	283 (55.6)
Private	224 (44)
Social security	2 (0.4)
Performance sector	
ICU	417 (81.9)
CRLMV	43 (8.4)
CCU	21 (4.1)
IMCU	11 (2.1)
Shock room	4 (0.7)
Other	13 (2.5)

ICU - intensive care unit; CRLMV – centers for rehabilitation and liberation from mechanical ventilation; CCU – cardiac care unit; UCIM - intermediate care unit. Values are expressed as numbers and percentages (%).

A total of 77.6% of the respondents reported using PC-CSV as the preferred ventilatory modality to initiate the partial support phase. Moreover, most of the respondents (356/509) consider the Richmond Agitation Sedation Scale (RASS) score to decide when to initiate PC-CSV, and of them, 320 (89.9%) consider adequate a score ≥ -3. Other general considerations related to PC-CSV use are shown in table 2. The subset of subjects who consider necessary an obligated PC-CSV period prior to performing a spontaneous breathing trial (SBT) was 42.8% (n = 218).

**Table 2:** General considerations related to the use of PC-CSV

Initial mode of the partial support phase	
PC-CSV	395 (77.6)
PC-CMV	62 (12.2)
VC-CMV	28 (5.5)
PAV+	17 (3.3)
Other	7 (1.4)
Utilities of the PC-CSV mode	500 (98.2)
Phase of partial support	349 (69.8)
Progressive reduction in ventilatory support	340 (68)
Spontaneous breathing trial	289 (57.8)
Consideration of RASS level to initiate PC-CSV	356 (70)
-5	4 (1.1)
-4	32 (9)
-3	109 (30.6)
-2	91 (25.6)
-1	73 (20.5)
0	47 (13.2)
Main advantage of the PC-CSV	
Avoid diaphragmatic atrophy	130 (25.5)
Improve comfort	130 (25.5)
Training the respiratory muscles	122 (24)
Decrease patient-ventilator dyssynchrony	118 (23.2)
Improve oxygenation	9 (1.8)

PS - inspiratory pressure support; PBW - predicted body weight; PC-CMV – pressure control-continuous mandatory ventilation; PC-CSV – pressure control-continuous spontaneous ventilation; VC-CMV – volume control-continuous mandatory ventilation; PAV+ - proportional assist ventilation plus. Values are expressed as numbers and percentages (%).

### PC-CSV settings and monitoring

A total of 223 participants select initial PS level based on a target Vt, while 402 choose the same initial PEEP level that the patient had during mandatory ventilation. The inability of the patient to remain under PC-CSV is determined mainly based on clinical variables of WOB, followed by Vt < 6mL/kg of predicted body weight (PBW) [n = 204 (40.1%)], a need for PS > 15cmH_2_O [n = 185 (36.3%)], RR > 25 [n = 180 (35.4%)] and Vt > 8mL/kg of PBW [n = 51 (10%)]. Regarding the cycling off criteria, 47% adjust it to adapt neural inspiratory time (Ti) to mechanical Ti, and 34% initiate at 25% of the peak inspiratory flow. In addition, 61 (12%) and 22 (2.8%) respondents choose a cycling off criterion of 30% and 50%, respectively, while 50 (4.3%) subjects do not consider it relevant. Other setting and monitoring variables can be observed in Table 3.

**Table 3:** Settings and monitoring

Selection of the initial PS based on	
Tidal volume 6 - 8mL/kg of PBW	223 (43.8)
WOB according to clinical evaluation	116 (22.8)
Respiratory rate	115 (22.6)
Pre-programmed value in PC-CMV	22 (4.3)
Plateau pressure monitored in VC-CMV	17 (3.3)
Advanced monitoring	13 (2.6)
Peak pressure monitored in VC-CMV	3 (0.6)
Modification of PS level based on	
WOB according to clinical evaluation	207 (40.7)
Vt according to ml/kg of PBW	123 (24.2)
Respiratory rate	107 (21)
Arterial blood gases	33 (6.5)
Advanced monitoring	24 (47)
Other	9 (1.8)
Objective of PEEP in PC-CSV	
Obtain the lowest WOB	161 (31.6)
Improve the mechanics of RS	160 (31.4)
Improve oxygenation	109 (21.4)
Avoid weaning delays, selecting the lowest possible value	65 (12.8)
Other	14 (2.8)
Selection of initial PEEP level	
Same value as programmed in mandatory mode	402 (79)
Value lower than that programmed in mandatory mode	55 (10.8)
I do not consider the value set in mandatory mode	38 (7.5)
Higher value than that programmed in mandatory mode	14 (2.8)

PS - inspiratory pressure support; PBW - predicted body weight; PC-CMV – pressure control-continuous mandatory ventilation; PC-CSV - pressure control-continuous spontaneous ventilation; VC-CMV - volume control-continuous mandatory ventilation; WOB - work of breathing; RS - respiratory system. Values are expressed as numbers and percentages (%).

Among the tools considered as ideal for monitoring the assistance provided, 55.2% (n = 281) prefer esophageal manometry, 10.8% (n = 55) P_0.1_, 8.3% (n = 42) diaphragmatic thickness fraction, 6.5% (n = 33) end-tidal partial pressure of CO_2_ (EtCO_2_), and 4.9% (n = 25) prefer the pressure muscular index, while 14.4% (n = 73) reported none of the available options.

### Over-assistance and under-assistance

**Figure 2:**
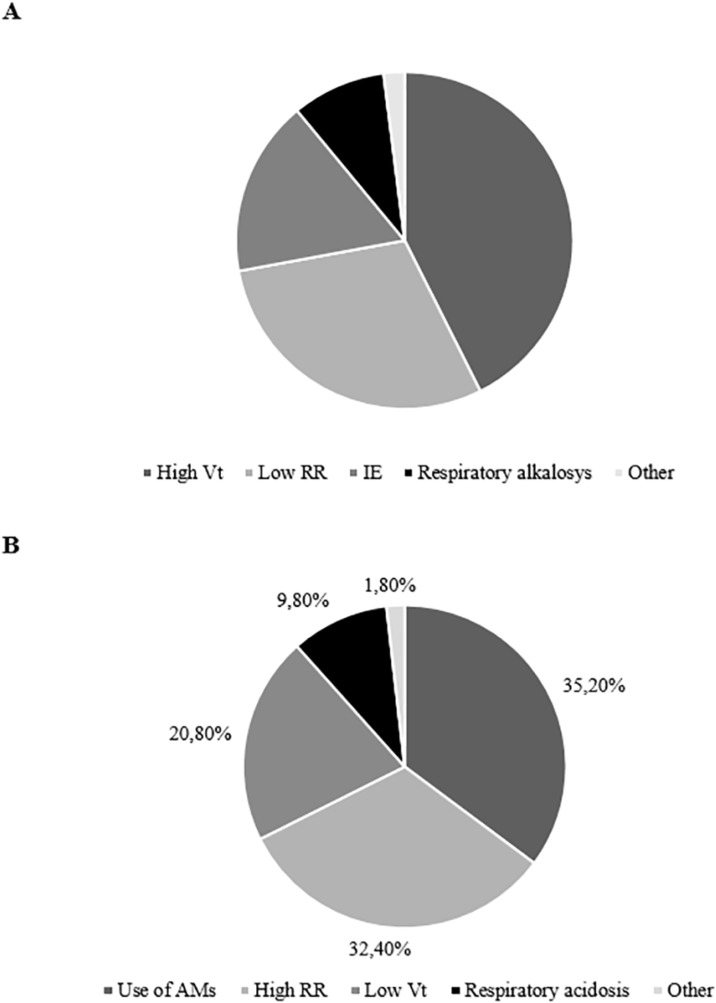
Percentage of use of clinical variables and monitoring for the detection of over-assistance and under-assistance A) Over-assistance criteria; B) Underassistance criteria. Vt - tidal volume; RR - respiratory rate; IE - ineffective efforts; AMs - accessory muscles.

When evaluating the diagnostic criteria of over-assistance and under-assistance, we found that high Vt and the use of accessory respiratory muscles (AMs) were respectively the most commonly selected (Figure 2).

Besides, we report the proportion of subjects who initially titrated PS based on RR, clinical variables or Vt, and choose the same variable to detect over- and under-assistance. Most frequently, those who chose them as initial criteria to set PS, use the same variable to later identify excessive or insufficient ventilatory assistance compared to those who chose none since the beginning (Table 4).

**Table 4:** Over-assistance and under-assistance based on variables used to titrate initial inspiratory support

	Do you use RR to titrate the initial PS level?		p value
	Yes (n = 109)	No (n = 385)	
Under-assistance based on respiratory rate	57 (52.3)	105 (27.3)	< 0.001*
Over-assistance based on respiratory rate	66 (60.6)	82 (21.3)	< 0.001*
	Do you use Vt to titrate the initial PS level?		
	Yes (n = 220)	No (n = 274)	
Under-assistance based on tidal volume	63 (28.6)	38 (13.9)	< 0.001*
Over-assistance based on tidal volume	115 (52.3)	97 (35.4)	< 0.001*
	Do you use clinical variables to titrate the initial PS level?		
	Yes (n = 115)	No (n = 379)	
Under-assistance based on the use of accessory muscles	54 (47.0)	120 (31.7)	0.003*
Over-assistance based on ineffective efforts	23 (20.0)	58 (15.3)	0.23

RR - respiratory rate; PS - inspiratory pressure support; Vt - tidal volume. Values are expressed as numbers and percentages (%).

* p < 0.05.

### Response comparison according to profession

**Figure 3:**
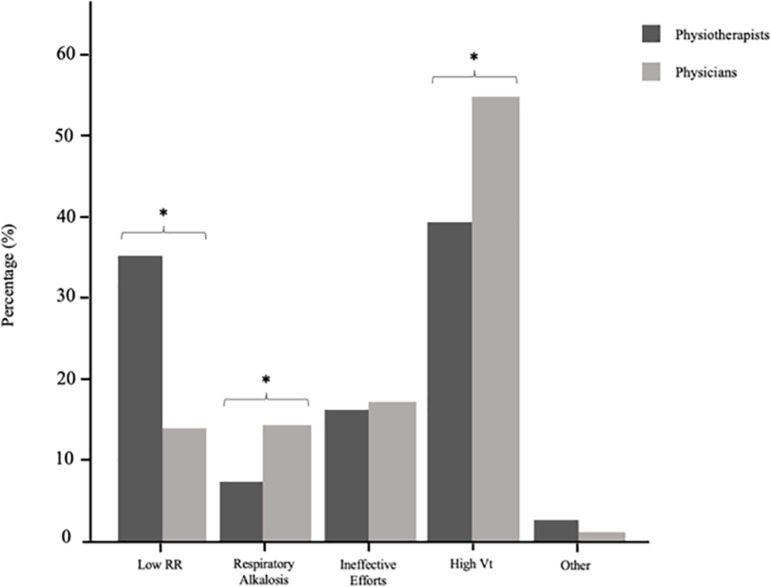
Identification of over-assistance according to physiotherapists and physicians. Vt - tidal volume. * p < 0.05.

**Figure 4:**
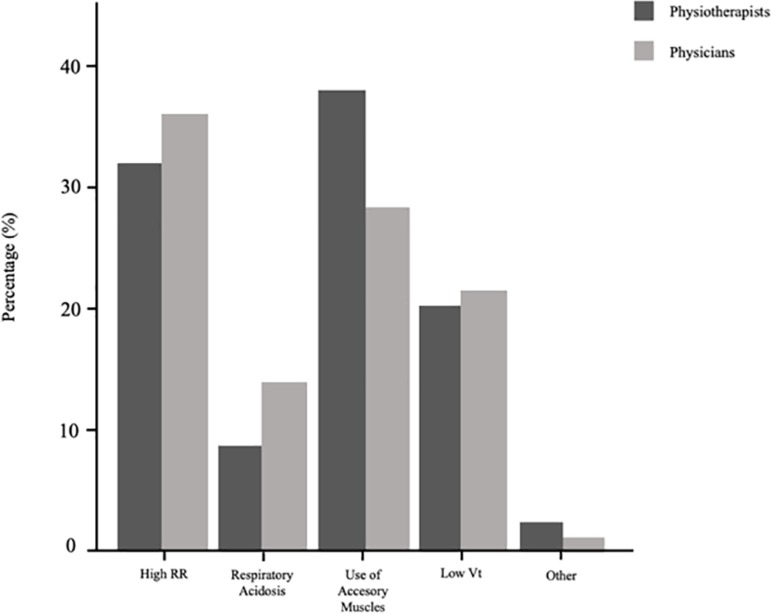
Identification of under-assistance according to physiotherapists and physicians. RR - respiratory rate; Vt - tidal volume.

Due to the small number of nurses in our sample (n = 15), their responses were not included for comparison among professions. The diagnostic criteria of over-assistance and under-assistance according to physicians and physiotherapists are presented in Figures 3 and 4. Statistically significant discrepancies were found between groups for identifying over-assistance, including: low RR (p < 0.001), respiratory alkalosis (p = 0.029) and high Vt (p = 0.003). There were no differences between groups regarding under-assistance criteria.

## DISCUSSION

The present study shows the results of the first survey to health care professionals in relation to the use and titration of PC-CSV in Argentina. The information obtained shows that PC-CSV is the most commonly used ventilatory mode during the partial support phase and is mostly used due to its advantages related to improving patient comfort and avoiding respiratory muscle atrophy.

### Titration of pressure support level

The three most selected variables both to initiate and modify the PS level were Vt, RR and clinical variables of WOB (use of AMs). Each of them deserves certain considerations.

### Tidal volume

Previous investigations have reported that Vt remains relatively constant during PC-CSV even with marked increase in WOB.^(9,11,12,18-20)^ Conversely, other studies have shown marked changes in Vt as WOB increases.^(21-24)^ Such discrepancies in these findings could be linked to the interpretation of the equation of motion of the respiratory system (RS):

$$P_{\mathrm{vent}}+P_{\mathrm{musc}}=E_{\mathrm{sr}}\;x\;∆V+R_{\mathrm{sr}}\;x\;V^{\prime }+{\mathrm{PEEP}}_{t}$$

where P_vent_ is the pressure generated by the ventilator, P_musc_ is the pressure exerted by the respiratory muscles, V’ is the inspiratory flow and R_sr_ and E_sr_ are RS resistance and elastance, respectively.

First, the evaluation methodology varies widely among studies. In cases where no changes in Vt are observed when WOB increases, the PS level used is variable, as well as the loading conditions to which the participants are subjected (e.g., continuous positive airway pressure - CPAP, flow by, minimum PS). Taking this into account, the muscular response will fundamentally depend on the PS provided by the ventilator because, by keeping the variables at the right of the equation relatively constant, if ventilatory assistance is very high, P_musc_ will decreases to maintain the equilibrium between the terms on each side of the equation.^(23,25)^ In absence of patient's effort, the Vt obtained will be a direct result of the formula: $\left(P_{\mathrm{aw}}-{V^{\prime }}_{\mathrm{cc}}\;x\;R_{\mathrm{sr}}\right)/E_{\mathrm{sr}}$, where P_aw_ is airway pressure and V’_cc_ is the cicling off air flow threshold. Considering this, in PC-CSV, it is possible to obtain a minimum Vt even at a muscle pressure of 0 cmH_2_O because the final Vt will not depend only on the intensity of the contraction of the respiratory muscles but also on their interaction with the aforementioned variables.^(10,23,25,26)^

On the other hand, in those studies where WOB changes are reflected in changes in Vt, the level of PS remains constant during the evaluation conditions and the inspired CO_2_ is intentionally modified in order to increase respiratory drive, making the muscular response almost exclusively dependent on chemical feedback and less influenced by the level of pressure support.^(21-24)^ However, it is necessary to consider that, due to the operational characteristics of the mode, the neuro-ventilatory coupling in PC-CSV is not perfect. This means that when changes in WOB occur, the Vt changes do not follow a linear relationship as they do during proportional modes.^(23,25)^

In summary, Vt is highly dependent on other variables beyond WOB, such as PS, cycling-off criterion and RS mechanics; therefore, it is necessary to consider each of them to correctly interpret the effectiveness of Vt as a titration parameter to adjust ventilatory support.

### Respiratory rate

In a recent study carried out by Plestch Asuncao et al., RR obtained the best positive predictive value, negative predictive value and area under the receiver operating characteristic (ROC) curve for detecting over- and under-assistance.^(9)^However, some previous studies question the validity of RR as an appropriate parameter for PS titration.^(11,13,21,25)^ Banner et al. studied the ability of a clinical score to infer WOB at different levels of PS, including RR as a variable observed in a sample of patients with acute respiratory failure. RR only explained 22% of the variance in the WOB, and the correlation between RR-WOB was 0.47.^(12)^ Nathan et al. and Brochard et al. studied 2 cohorts of patients in the weaning process during different SBT modalities and evaluated WOB and the pressure-time product (PTPi) of the respiratory muscles. Besides, they monitored changes in different variables, including RR. In both studies, WOB and PTPi increased as the load imposed by the ventilatory modality increased; while the RR was not sensitive to changes in WOB, within a variation lower than 4 breaths per minute (bpm) between the most and least demanding conditions.^(19,27)^ This findings are consistent with those reported by other authors where manipulation of PCO_2_ over a wide range from moderate hypocapnia to mild hypercapnia has no appreciable effect on RR behavior.^(23)^ Moreover, other studies have shown that, in some cases, the decrease in RR with an increase in PS is in fact fictitious because as hypocapnia is deepened, ineffective efforts (IE) are more frequent.^(28)^

Therefore, the “ventilator RR” should be taken with caution as a surrogate of “patient RR” because ignoring the presence of IE could increase the risk of over-assistance in PC-CSV.

### Clinical variables of WOB

The clinical change in patient´s ventilatory pattern are included as defining criteria of SBT failure or as indicator to increase ventilatory assistance.^(9,29,30)^ Within these clinical parameters, the intensity of contraction of the sternocleidomastoid muscle (SCM) is probably the most studied in IMV.^(20)^

Brochard et al. studied a cohort of 8 patients with prolonged weaning criteria, 4 of whom had chronic obstructive pulmonary disease (COPD) and observed that the optimal PS corresponded to the point at which the SCM electromyographic activity decreased to a minimum. Based on this, they propose titrating PS through muscle palpation and selecting a value immediately above the point where SCM contractile activity begins to be noticeable.^(20)^ In line with these findings, Perrigault et al. observed changes in P_0.1_ only when SCM electromyographic activity was observed. No other ventilation parameter significantly inferred changes in P_0.1_. The study suggests P_0.1_ < 2.9cmH_2_O as a cutoff to avoid the use of AMs and the predisposition to fatigue.^(31)^ Interestingly, some studies note that the SCM activation starts at inspiratory effort levels close to 35 - 40% of the maximum inspiratory pressure, which coincides with threshold values previously proposed to avoid the development of diaphragmatic fatigue.^(32-35)^

Therefore, the inspiratory activity of AMs, particularly the SCM, should be considered as an additional clinical monitoring variable when selecting inspiratory pressure support in PC-CSV.

### Titration of PEEP in PC-CSV

There is limited information on how to select the appropriate PEEP level in spontaneously breathing patients under IMV. Most of our sample reported choosing this value with the aim to decrease WOB and improve RS mechanics. Theoretically, these two objectives should be closely related because, if the RR and Vt remain constant, any modification in the mechanical properties of the RS that leads to lower driving pressure need will unfailingly translate to a reduced WOB.

Using animal models and a cohort of patients with acute respiratory distress syndrome (ARDS), Morais et al. observed a decrease in esophageal pressure (Pes) and transpulmonary pressure (Ptp) swings when applying PEEP values of 15cmH_2_O compared to 5cmH_2_O. One of the potential explanations for these effects was the improvement in RS mechanics resulting from alveolar recruitment. However, the Vt of the group of subjects with ARDS also decreased; therefore, RS compliance may not have improved.^(36)^Some alternative explanations for the reduction in WOB could be the activation of receptors related to the Hering Breuer reflex, the homogenization of the gas distribution among different areas of the lung and the modification of neuromechanical coupling, leading to diaphragmatic mechanical disadvantage with the consequent reduction in its force generation capacity.^(36,37)^

In 13 patients with COPD under IMV in the partial support phase, MacIntyre et al. analyzed the gradual application of external PEEP and observed a decrease in intrinsic PEEP and PTPi associated with intrinsic PEEP without changes in Vt. Similarly, Petrof et al. examined the effects of external PEEP application on WOB, ventilatory pattern and dyspnea in 7 patients with COPD during weaning from IMV and observed a significant decrease in Pes and Ptp swings and a reduction of up to 50% of the total WOB with increases in PEEP up to 15 cmH_2_O. All patients reported a clinical improvement of dyspnea.^(38,39)^

In summary, although it seems clear that the application of PEEP in patients with COPD during spontaneous breathing should aim to reduce the intrinsic PEEP considering the dynamic collapse and avoiding alveolar overdistention, further studies are required to elucidate the effect of PEEP on WOB in patients with ARDS.

### Over-assistance

The different diagnostic criteria reported among professionals to define over-assistance could be explained by the scarce attention paid to this phenomenon in clinical practice as well as the inherent difficulties regarding its recognition. The “over-assisted” patient usually seems calm and comfortable, which could lead to underestimating its associated complications.^(9)^However, elevated PS levels can generate hyperinflation, respiratory alkalosis, diaphragmatic atrophy, a depression of respiratory drive with consequent apnea and IE which may be reflected in periodic and alternating ventilatory pattern.^(9,10)^ Using WOB and central drive measurement variables, Plestch Assuncao et al. generated the first formal definition of over-assistance in the literature. Interestingly, in their study, using baseline PS levels of only 8cmH_2_O, 37 - 48% of the patients presented over-assistance criteria, a percentage that increased to 90% with PS values of 17 - 20cmH_2_O.^(9)^In our survey, the most widely used variable for diagnosing over-assistance was Vt, which does not correspond to the previously proposed definitions. These discrepancies could be explained by the limited use of esophageal pressure monitoring as it was observed in the LUNG SAFE study, although more than 50% of our sample identified this tool as ideal for estimating WOB.^(40)^

### Under-assistance

The adverse consequences of inadequate ventilatory support have been extensively detailed, mainly in terms of diaphragmatic injury, progression of the degree of lung injury, deterioration of oxygenation/gas exchange and hypercapnia.^(14,25,35)^

In our survey, the use of AMs and high RR were the two preferred parameters for diagnosing under-assistance. Additionally, there were no discrepancies between physicians and physiotherapists in relation to the detection criteria, which reflects the importance assigned to these parameters as classic signs of inspiratory muscle fatigue. However, we must consider that when PS is reduced, a RR as high as 35 - 45bpm may not necessarily indicate an elevated respiratory drive or an increase in the ventilatory demand but represent the patient’s preferred “non-stressed” triggering frequency or reflect the elimination of IE that now are observed as effective triggered cycles. Non-stressed RR is widely variable between individuals and is usually, on average, 10bpm higher in critically ill patients.^((26.28))^ In turn, an increase in RR will decrease the inspiratory (Ti) and expiratory (Te) time, keeping the Ti/Te ratio constant to ensure proper diaphragmatic perfusion.^(21,22,28)^ In this sense, the Ti and total time ratio (Ti/Ttot), contextualized in the transdiaphragmatic pressure (Pdi) relative to its maximum possible (Pdi_MAX_), is an important determinant of the ability to maintain effort over time. Therefore, using the tension-time index (TTI = Pdi/Pdi_MAX_ x Ti/Ttot) could represent a more adequate measure to determine the fatiguing effect of an elevated RR.^(34)^

## CONCLUSION

PC-CSV was the most commonly used ventilatory mode for the partial ventilatory support phase. The most frequently chosen variable to titrate inspiratory pressure support level was tidal volume, and the main objective of PEEP selection was to decrease work of breathing. Over-assistance was detected primarily by high tidal volume, while under-assistance was detected by the use of accessory muscles. Statistically significant differences where only found between physiotherapists and physicians in diagnostic criteria for over-assistance, not so for under-assistance.

## Appendix 1

**Appendix 1:** Survey on the use and titration of PC-CSV in Argentina

**1) How old are you?**
Less than 24 years
Between 25 and 34 years
Between 35 and 44 years
Between 45 and 50 years
More than 50 years

**2) What is your profession?**
Physician
Physiotherapists
Nurse

**3) How many years of experience do you have in the care of critical patients?**
Less than 5 years
Between 5 and 9 years
Between 10 and 14 years
Between 15 and 19 years
More than 20 years

**4) In which health system do you work the most hours per week?**
Public
Social security
Private

**5) In what area do you mostly perform your healthcare practice?**
Intensive care unit (ICU)
Cardiac care unit (CCU)
Intermediate care unit (IMCU)
Shock room
Centers for rehabilitation and liberation from mechanical ventilation (CRLMV)
Other (specify)

**6) In your usual practice, what ventilatory mode do you select to INITIATE the partial ventilatory support phase (the moment at which the patient begins to voluntarily activate the respiratory muscles)?**
Volume controlled ventilation (VC-CMV)
Pressure controlled ventilation (PC-CMV)
Pressure support ventilation (PC-CSV)
Proportional assist ventilation plus (PAV+)
Other (specify)

**7) In which of the following scenario/s do you use the PC-CSV? (more than one answer can be chosen)**
Partial ventilatory support phase
As a method for the gradual reduction in ventilatory support
As a modality for the spontaneous breathing trial
I do not usually use this ventilatory mode

**8) What level of the RASS (Richmond Agitation Sedation Scale) do you consider adequate to start using PC-CSV?**
-5 (no response to physical stimulation)
-4 (responds only to physical stimulation)
-3 (eye opens to voice, but no eye contact)
-2 (eye opens to voice with eye contact for less than 10 seconds)
-1 (eye opens to voice with eye contact for more than 10 seconds)
0 (alert and calm)
I do not consider the RASS level

**9) Do you consider it necessary the patient to remain in PC-CSV for a period of time before performing a spontaneous breathing trial?**
Yes
No

**10) What is the main advantage for which you use PC-CSV?**
Improve patient comfort
Decrease patient - ventilator asynchrony
Avoid diaphragmatic atrophy
Training of respiratory muscles
Improve oxygenation

**11) In your common practice, how do you determine the INITIAL pressure support level when you start setting PC-CSV?**
I set the same pressure value programmed in pressure control mode
I set the peak pressure value monitored in volume control mode
I set the plateau pressure value monitored in volume control mode
I set the value that generates a Vt of 6 - 8mL/kg of predicted body weight
I set the value that results in an adequate respiratory rate
I set the value that generates the lowest WOB according to clinical evaluation
I set the value that generates the lowest WOB according to advanced monitoring (esophageal pressure, etc.)

**12) In your usual practice, how do you determine the PEEP level when you start setting PC-CSV?**
I set the same value that the patient had in mandatory mode
I set a lower value than the patient had in mandatory mode
I set a higher value than the patient had in mandatory mode
I do not consider the value programmed in mandatory mode

**13) Which is the MAIN objective you follow when setting the PEEP level in PC-CSV?**
To obtaining the best possible oxygenation
To avoid weaning delay, selecting the lowest possible PEEP
To obtain the lowest possible WOB
To improve the respiratory system mechanics

**14) What is the cycling-off criterion you choose when START using PC-CSV?**
25%
30%
50%
The one that allows adapting the mechanical inspiratory time to the neural inspiratory time
I do not consider it relevant

**15) Which of the following parameters do you consider to be MOST IMPORTANT to decide that an inspiratory pressure support level modification is necessary?**
The absolute pressure support level applied to the system (peak pressure)
Tidal volume according to mL/kg of predicted body weight
Respiratory rate
Work of breathing measured clinically (use of accessory muscles, etc.)
Work of breathing according to advanced monitoring (esophageal pressure, etc.)
Arterial blood gases

**16) On which variable/s do you MAINLY rely for considering the patient is not able to remain in PC-CSV? (more than one answer can be chosen)**
The need to use pressure support levels greater than 15cmH_2_O
The patient´s tidal volume is lower than 6mL/kg
The patient´s tidal volumes is greater than 8mL/kg
The patient has a respiratory rate greater than 25 per minute.
Clinical variables (use of accessory muscles, diaphoresis, etc.)

**17) In your daily practice, which of the following variables do you consider to diagnose ventilatory over-assistance in PC-CSV?**
High tidal volume
Low respiratory rate
Presence of ineffective efforts
Presence of respiratory alkalosis

**18) In your daily practice, which of the following variables do you consider to diagnose under-assistance in PC-CSV?**
Low tidal volume
High respiratory rate
Use of accessory muscles
Presence of respiratory acidosis

**19) Regardless of availability in your daily practice, which of the following tools do you consider ideal to monitor the degree of ventilatory support given?**
Esophageal pressure (Pes swing, PTP, Campbell diagram)
Diaphragmatic thickness fraction (ultrasound)
End-tidal CO_2_ (EtCO_2_)
P0.1
Pressure muscular index (PMI)
None
